# Assessing the Influence of Indoor Exposure to “Outdoor Ozone” on the Relationship between Ozone and Short-term Mortality in U.S. Communities

**DOI:** 10.1289/ehp.1103970

**Published:** 2011-11-18

**Authors:** Chun Chen, Bin Zhao, Charles J. Weschler

**Affiliations:** 1 Department of Building Science, School of Architecture, Tsinghua University, Beijing, China; 2 Environmental and Occupational Health Sciences Institute, University of Medicine and Dentistry of New Jersey (UMDNJ)–Robert Wood Johnson Medical School and Rutgers University, Piscataway, New Jersey, USA; 3 International Centre for Indoor Environment and Energy, Technical University of Denmark, Lyngby, Denmark

**Keywords:** air change rate, air conditioning, infiltration rate, outdoor-to-indoor transport, ozone-derived products, total exposure

## Abstract

Background: City-to-city differences have been reported for the increase in short-term mortality associated with a given increase in ozone concentration (ozone mortality coefficient). Although ozone concentrations are monitored at central outdoor locations, a large fraction of total ozone exposure occurs indoors.

Objectives: To clarify the influence of indoor exposure to ozone of outdoor origin on short-term mortality, we conducted an analysis to determine whether variation in ozone mortality coefficients among U.S. cities might be partly explained by differences in total ozone exposure (from both outdoor and indoor exposures) resulting from the same outdoor ozone concentration.

Methods: We estimated average annual air change rates (the overall rate at which indoor air is replaced with outdoor air) and used these to estimate the change in total ozone exposure per unit change in outdoor ozone exposure (ozone exposure coefficient) for 18 cities that had been included in the National Morbidity and Mortality Air Pollution Study (NMMAPS). We then examined associations between both parameters and published ozone mortality coefficients.

Results: For the 18 targeted NMMAPS cities, the association between ozone mortality coefficients and ozone exposure coefficients was strong (1-hr ozone metric: *R^2^* = 0.58, *p* < 0.001; 8-hr ozone: *R^2^* = 0.56, *p* < 0.001; 24-hr ozone: *R^2^* = 0.48, *p* = 0.001). When extended to another 72 NMMAPS cities, the associations remained strong (*R^2^* = 0.47–0.63; *p* < 0.001).

Conclusions: Differences in ozone mortality coefficients among cities appear to partially reflect differences in total ozone exposure resulting from differences in the amount of outdoor ozone that is transported indoors.

Numerous epidemiological studies have shown an association between an increase in outdoor ozone concentration and an increase in short-term mortality (e.g., Bell et al. 2004, 2005; Gryparis et al. 2004; Hubbell et al. 2005; Ito et al. 2005; Levy et al. 2005; Parodi et al. 2005; Smith et al. 2009; Zhang et al. 2006). However, in different cities the same increase in the concentration of outdoor ozone may result in different increases in total ozone exposures (the sum of outdoor and indoor exposures) because indoor exposure varies with the rate at which indoor air is replaced with outdoor air (the air change rate) for buildings within each city and the amount of time that residents spend indoors (Weschler et al. 1989). Bell et al. (2004) estimated the percent increase in short-term mortality per 10-ppb increase in ozone (ozone mortality coefficient) for 95 U.S. urban communities based on data from the National Morbidity, Mortality, and Air Pollutions Study (NMMAPS). Their point estimates for ozone mortality coefficients ranged from –0.2% for Orlando, Florida, to 1.7% for New York, New York. Weschler subsequently suggested that differences in ozone mortality coefficients among cities could be partially explained by differences in outdoor-to-indoor transport of ozone (see Table 3 of Weschler 2006). Bell and Dominici (2008) examined whether heterogeneity in ozone mortality coefficients could be explained by differences in community-specific characteristics, and identified a higher prevalence of central air conditioning (AC) as one of several factors associated with reduced ozone-related mortality.

Smith et al. (2009) reassessed the relationship between ozone and short-term mortality for the NMMAPS urban communities, including an investigation of alternative ozone exposure metrics—namely, daily maximum 8-hr or 1-hr averages, as alternatives to the 24-hr average ozone levels used by Bell et al. (2004). They also examined regional influences on ozone mortality coefficients, as well as between-city effect modifiers. Consistent with the findings of Bell and Dominici (2008), Smith et al. (2009) found the prevalence of central AC was inversely associated with ozone-related mortality. However, they also found a stronger positive association between ozone-related mortality and the prevalence of air conditioners installed in a window (window AC).

Using construction characteristics from a set of 209 dwellings representing different types of homes, Persily et al. (2010) modeled frequency distributions of residential infiltration rates (the rate at which a given building’s air is replaced with outdoor air when its windows are closed) in 19 cities representing a range of U.S. climatic conditions. For each city, the authors estimated hour-by-hour infiltration rates over a typical weather year for each of the 209 house types (including detached homes, attached homes, manufactured homes, and apartments) that were further characterized by year built, number of floors, foundation type, central AC, and other characteristics relevant to indoor air quality. The resulting data were combined using weighting factors that accounted for the fraction of each house type in each city.

The reanalysis by Smith et al. (2009) of the association between ozone and short-term mortality in the NMMAPS cities, and the availability of the detailed estimates of infiltration rate distributions in representative U.S. cities by Persily et al. (2010), prompted us to reexamine the hypothesis that differences in ozone mortality coefficients among the NMMAPS cities can be partially explained by differences in outdoor-to-indoor transport and the resulting total ozone exposures. Our specific aim was to examine the relationship between total ozone exposure—accounting for indoor exposure—and ozone mortality coefficients for NMMAPS cities whose infiltration rates have been modeled by Persily et al. (2010) and then to extend the analysis to an additional 72 NMMAPS urban communities. To our knowledge, this is the first time that the relationship between total ozone exposure and ozone mortality coefficients has been examined for a large set of cities.

## Methods

**Figure 1:**
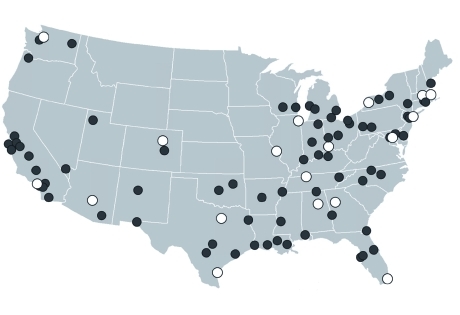
Location of the 18 NMMAPS cities for which detailed modeled infiltration rates were available (open circles) and the 72 additional NMMAPS cities included in the extended analysis (filled circles).

*Cities.* We initially focused on 18 U.S. cities that were included in the NMMAPS study and were selected by Persily et al. (2010) to represent different climatic regions of the United States. We then extended the analysis to 72 additional NMMAPS cities with climatic conditions and housing stock similar to one of the 18 cities used in the original analysis (Figure 1).

*Average annual infiltration rates.* The infiltration rate is the rate at which a given building’s air is replaced with outdoor air when its windows are closed. We began with the infiltration rate distributions published in Table 8 of Persily et al. (2010), reported for each city as the percent of hours that residences had infiltration rates below 0.25, 0.35, 0.5, 0.75 and 1.0 per hour. The average annual infiltration rate for a given city (λ_infilt_) was estimated from these data by assuming that the infiltration rates among the residences in a given city were log-normally distributed, an assumption supported by a number of studies (e.g., Bekö et al. 2010; Murray and Burmaster 1995). For each city, we plotted a cumulative frequency distribution between the *z*-score for the percent of hours residences were below a given infiltration rate and the natural log of that infiltration rate. Based on the resulting linear relationship, we estimated λ_infilt_ for each of the 18 targeted NMMAPS cities.

*Fraction of time cooling occurs.* We assumed that the fraction of time that cooling occurs (*x*) corresponded to the fraction of time that AC operated in residences with central AC, and the fraction of time that windows were open in residences without central AC. In a given city, *x* was estimated using a method based on the monthly maximum and minimum temperature, *T_max_* and *T_min_*, throughout a statistical year as reported by the National Oceanic and Atmospheric Administration (NOAA 2002). In this approach, we assumed that cooling occurred if the temperature was higher than 24°C. We focused on cooling rather than heating because of the higher ozone levels in summer. [For further details, see Supplemental Material, p. 2 (http://dx.doi.org/10.1289/ehp.1103970)].

*Fraction of residences with central AC.* We used estimates for the fraction of residences with central AC (*y*) from Smith et al. (2009 and personal communication). For the cities in common, these estimates agree reasonably well with those reported for 36 U.S. cities (see Table 3 of Medina-Ramon et al. 2006), indicating that different research groups have arrived at similar city-specific estimates for this parameter.

*Average annual air change rate.* The air change rate (λ_overall_) is the overall rate at which a given building’s air is replaced with outdoor air. Whereas λ_infilt_ represents the rate of air change when windows are closed, λ_overall_ also accounts for the additional air change that occurs when windows are open. As a first approximation, we assumed that window-opening only occurred in residences without central AC. Based on this assumption, we derived the following equation:

λ_overall_ = λ_infilt_ + (*x*)(1–*y*) λ_open_, [1]

where λ_open_ is the difference in air change rate in residences without central AC when windows are open versus closed. [See Supplemental Material, pp. 2–3 (http://dx.doi.org/10.1289/ehp.1103970), for details regarding the derivation of Equation 1.] We assumed a value of 1.5/hr for λ_open_ based on results presented by Alevantis and Girman (1989). The sensitivity of our results to this parameter, which is difficult to estimate, is examined later in this paper.

This estimate of λ_overall_ does not account for window opening on mild days or window opening in homes with central AC as an alternative to AC operation.

*Changes in indoor ozone and ozone-derived products.* The change in indoor ozone concentration per 10-ppb change in outdoor ozone, Δ[O_3_]_in_, can be approximated by the following equation:

Δ[O_3_]_in_ = [λ_overall_/(λ_overall_ + *k*_sr_)] 10 ppb, [2]

where *k*_sr_ is the first order rate constant for ozone removal by indoor surfaces (Weschler 2000, 2006; Weschler et al. 1989).

In addition to indoor ozone that is directly transported from outdoors, we also need to account for exposure to ozone oxidation products that form indoors. The change in ozone-derived products per 10-ppb change in outdoor ozone, Δ[prod]_in_, can be approximated by the following equation:

Δ[prod]_in_ = [(*yld_g_* (*k*_sr_) ÷ (λ_overall_ + *k*_sr_)] 10 ppb, [3]

where *yld_g_* is the yield of gas phase products resulting from surface reactions. [For details regarding the derivation of Equation 3, see Supplemental Material, p. 3 (http://dx.doi.org/10.1289/ehp.1103970).] Estimates for Δ[O_3_]_in_ and Δ[prod]_in_ were calculated using Equations 2 and 3, with *k*_sr_ = 3.0/hr (Weschler 2000) and *yld_g_* = 0.3 (Weschler 2006).

*Ozone exposure coefficients.* We define “exposure” in a specific microenvironment as the product of “time spent in the microenvironment” and “pollutant concentration in the microenvironment during that time.” The change in total ozone exposure is the sum of the change in ozone exposure in each of the microenvironments that a person spends a fraction of their time, and it is approximated as the sum of the changes in outdoor and indoor exposures. The change in total ozone exposure per unit change in outdoor ozone exposure, ΔO_3_exposure_, is given by the following equation:

ΔO_3_exposure_ = 1+ (*t*_in_/*t*_out_) × [λ_overall_/(λ_overall_ + *k*_sr_)]. [4]

[See Supplemental Material, p. 4 (http://dx.doi.org/10.1289/ehp.1103970), for details regarding the derivation of Equation 4.] Throughout this manuscript we refer to “ΔO_3_exposure_” as the ozone exposure coefficient. For the ratio of time spent indoors to time spent outdoors and in vehicles (*t*_in_/*t*_out_), we used region-specific values from the National Human Activity Pattern Survey (NHAPS) (Klepeis et al. 2001).

*Ozone mortality coefficients.* Ozone mortality coefficients correspond to the percent increase in short-term mortality per 10-ppb increase in outdoor ozone. Bell et al. (2004) calculated ozone mortality coefficients based on 24-hr ozone using a hierarchical Bayesian method. In addition to 24-hr ozone, Smith et al. (2009) calculated ozone mortality coefficients based on daily maximum 1-hr and 8-hr ozone levels. In addition to “national prior” estimates, Smith and coworkers calculated “regional prior” estimates using a more general version of the random effects model that accounted for regional differences in covariates. City-specific ozone mortality coefficients were sensitive to which of the two forms of “prior” was chosen. We used the “regional prior” ozone mortality coefficients from Figures 1, 4, and 5 of Smith et al. (2009) [see Supplemental Material, Table 1 (http://dx.doi.org/10.1289/ehp.1103970)]. The variation of the ozone mortality coefficients with ozone metric is discussed in the Supplemental Material (p. 5).

**Table 1:** Key parameters and calculated results for 18 NMMAPS cities with published infiltration rate distributions (Persily et al. 2010).

Parameters									Calculated results								
No.	City	Population growth 1990–2000*a* (%)	λ_infilt_**(hr^–1^)	CDD	*x*	*y*	λ*overall *(hr^–1^)	Δ[*O3*]_in _(ppb)	Δ[prod]_in _(ppb)	*tin*/*tout*	ΔO_3_exposure_
1		Atlanta, GA		5.7		0.43		1,006		0.22		0.86		0.48		1.4		2.6		6.47		1.89
2		Birmingham, AL		–8.7		0.43		1,045		0.22		0.80		0.50		1.4		2.6		6.47		1.92
3		Boston, MA		2.6		0.68		432		0.067		0.18		0.76		2.0		2.4		7.58		2.54
4		Buffalo, NY		–10.8		0.70		304		0.033		0.43		0.73		2.0		2.4		6.74		2.32
5		Chicago, IL		4.0		0.61		464		0.067		0.51		0.66		1.8		2.5		6.83		2.23
6		Cincinnati, OH		–9.0		0.52		672		0.11		0.57		0.59		1.6		2.5		6.83		2.12
7		Corpus Christi, TX		7.8		0.48		1,943		0.41		0.78		0.62		1.7		2.5		6.58		2.12
8		Dallas/Ft. Worth, TX		18.0		0.50		1,428		0.32		0.89		0.55		1.6		2.5		6.58		2.02
9		Denver, CO		18.6		0.49		386		0.092		0.32		0.58		1.6		2.5		6.54		2.07
10		Los Angeles, CA		6.0		0.42		837		0.020		0.34		0.44		1.3		2.6		6.46		1.83
11		Miami, FL		1.1		0.35		2,435		0.59		0.80		0.53		1.5		2.6		6.47		1.97
12		Nashville, TN		11.7		0.51		920		0.18		0.83		0.56		1.6		2.5		6.47		2.01
13		New York City, NY		9.4		0.62		644		0.10		0.10		0.76		2.0		2.4		6.74		2.36
14		Phoenix, AZ		34.3		0.42		2,327		0.44		0.92		0.47		1.4		2.6		6.46		1.88
15		Seattle, WA		9.1		0.62		107		0.0		0.06		0.62		1.7		2.5		5.61		1.96
16		St. Louis, MO		–12.2		0.58		867		0.23		0.80		0.65		1.8		2.5		6.27		2.11
17		Washington, DC		–5.7		0.54		867		0.12		0.82		0.57		1.6		2.5		6.66		2.07
18		Worcester, MA		1.7		0.60		206		0.027		0.10		0.64		1.8		2.5		7.58		2.33
Abbreviations: λ_infilt_, average infiltration rate when windows are closed; CDD, cooling degree days; *x*, fraction of year cooling occurs; *y*, fraction of residences with central AC; λ_overall_, average overall air change rate; Δ[O_3_]_in_, change in indoor concentration of ozone per 10-ppb change in outdoor ozone; Δ[prod]_in_, change in indoor concentration of ozone-derived products per 10-ppb change in outdoor ozone; *t*_in_/*t*_out_, ratio of time spent indoors to time spent outdoors and in vehicles (from Table 10 of Klepeis et al. 2001); ΔO_3_exposure_, change in total ozone exposure per unit change in outdoor ozone exposure (i.e., the ozone exposure coefficient). **a**Population growth (US Census Bureau 2011) is a rough indicator for the average age of buildings in a city.																						

*Exploring correlations for other NMMAPS cities.* U.S. cities with a population > 250,000 have been paired with “representative” cities from among the original 18 for the purpose of modeling ventilation rates [see Table A1 of Vandemusser Design LLC (2007)]. From this listing, we selected an additional 72 NMMAPS cities for which we judged the “representative city” to adequately match the NMMAPS city in terms of climate. Each of these cities was assigned an average annual infiltration rate equal to that of its representative city. The fraction of time that cooling occurred (*x*) was calculated using the procedure described above. The fraction of residences with central AC (*y*) was taken from the data set provided by R.L. Smith (personal communication). “Overall air change rates” were then calculated using Equation 1 and ozone exposure coefficients were calculated using Equation 4. These values, as well as other key input parameters, are tabulated in Supplemental Material, Table 2 (http://dx.doi.org/10.1289/ehp.1103970).

**Table 2:** Coefficient of determination (*R*^2^) and *p*-value between various parameters in Table 1 and ozone mortality coefficients based on the 1-hr, 8-hr, or 24-hr ozone metric.

1-hr [O_3_]			8-hr [O_3_]			24-hr [O_3_]		
Parameter	*R2*	*p*	*R2*	*p*	*R2*	*p*
18 cities												
Overall air change rate		0.51		< 0.001		0.41		0.004		0.34		0.01
Ozone exposure coefficient		0.58		< 0.001		0.56		< 0.001		0.48		0.001
Fraction with central AC		0.28		0.023		0.09		0.24		0.09		0.22
90 cities												
Overall air change rate		0.43		< 0.001		0.50		< 0.001		0.45		< 0.001
Ozone exposure coefficient		0.47		< 0.001		0.63		< 0.001		0.54		< 0.001
Fraction with central AC		0.33		< 0.001		0.17		< 0.001		0.17		< 0.001


**Table 3:** Sensitivity of the association between ozone exposure coefficient and ozone mortality coefficient to values for key parameters.

1-hr [O_3_]			8-hr [O_3_]			24-hr [O_3_]		
Parameter	*R2*	*p*	*R2*	*p*	*R2*	*p*
*x* – default value		0.58		< 0.001		0.56		< 0.001		0.48		0.001
*x*_min_ – lower estimate		0.58		< 0.001		0.51		< 0.001		0.45		0.002
*x*_max_ – upper estimate		0.45		0.002		0.41		0.004		0.31		0.007
λ_open_ – 1.5/hr (default value)		0.58		< 0.001		0.56		< 0.001		0.48		0.001
λ_open_ – 0.5/hr (lower estimate)		0.61		< 0.001		0.56		< 0.001		0.49		0.001
λ_open_ – 5.0/hr (upper estimate)		0.29		0.022		0.34		0.011		0.27		0.026
*k*_sr_ – 3/hr (default value)		0.58		< 0.001		0.56		< 0.001		0.48		0.001
*k*_sr_ – 0.8/hr (lower estimate)		0.57		< 0.001		0.58		< 0.001		0.51		< 0.001
*k*_sr_ – 7.6/hr (upper estimate)		0.57		< 0.001		0.55		< 0.001		0.48		0.001
*y* – default value		0.58		< 0.001		0.56		< 0.001		0.48		0.001
*y* – upper estimate		0.48		0.001		0.48		0.001		0.46		0.002
*t*_in_*/t*_out_ – default value		0.58		< 0.001		0.56		< 0.001		0.48		0.001
*t*_in_*/t*_out_ – upper value		0.47		0.002		0.59		< 0.001		0.50		0.001
Abbreviations: *x*, fraction of year cooling occurs; λ_open_, difference in air change rate when windows are open versus when they are closed; *k*_sr_, rate constant for ozone removal by indoor surfaces; *y*, fraction of residences with central AC; *tin/tout*, ratio of time spent indoors to time spent outdoors and in vehicles.												

*Sensitivity analysis.* We examined the sensitivity of our results to values for key parameters by examining correlations between ozone mortality coefficients and ozone exposure coefficients when the latter were calculated with what we judged to be reasonable bounds for these parameters. The lower bound for the fraction of year cooling occurs, *x*, was based on estimates of total hours of AC compressor operation per year in various U.S. cities. These estimates are from the Engineering Documentation that is part of Lawrence Berkeley National Laboratory’s Home Energy Saver program (Appendix C, Local Climate Parameters; see https://sites.google.com/a/lbl.gov/hes-public/calculation-methodology/appendices/appendix-c). This method for estimating the fraction of time that cooling occurs is conservative because AC compressors only operate a fraction of the time that AC systems operate. The upper bound estimates were calculated by an approach analogous to that used to calculate the default value of *x*; however, rather than assuming that cooling occurred at temperatures higher than 24°C, we assumed that cooling occurred at temperatures higher than 18.3°C. This is the base temperature used by NOAA in their calculation of cooling degree days (CDDs), a unit that relates daily temperature to AC demand. This method likely results in an overprediction because few people operate AC systems when the temperatures are between 18.3°C and 24°C. To bound the difference in the air change rate when windows are open versus when they are closed, λ_open_, we chose 0.5/hr as a lower estimate and 5.0/hr as an upper estimate. These selected lower and upper limit estimates are based on a study of window opening and the subsequent measured air change rates by Alevantis and Girman (1989). We have bounded the estimate of the first order rate constant for ozone removal by indoor surfaces, *k*_sr_, based on studies summarized in Table 3 of Weschler (2000). The upper estimate for this parameter is 7.6/hr, while the lower estimate is 0.8/hr. The upper estimate for the fraction of residences with central AC, *y*, was simply the value for the fraction of residences with any type of AC (central or window AC). To examine the sensitivity of the present analysis to *t*_in_*/t*_out_, we treated time in vehicles as either “outdoor time” or “indoor time.” The values for *t*_in_*/t*_out_ were calculated using data presented in Table 10 of Klepeis et al. (2001). Both the default values and the upper bound values for *t*_in_*/t*_out_ are listed in Supplemental Material, Table 3.

## Results

*Average annual air change rate.* Table 1 presents average annual infiltration rates (λ_infilt_) and overall air change rates (λ_overall_) for housing in the 18 NMMAPS cities that are the focus of the present study. Values for λ_infilt_ represent air change rates when windows are closed, whereas values for λ_overall_ additionally account for periods when windows are open. Infiltration rates are largely influenced by air leakage across exterior walls, and older residences tend to have leakier exterior walls (Persily et al. 2010). Hence cities with a larger fraction of older residences (e.g., Boston, Buffalo, Worcester) tend to have higher λ_infilt_ values. In addition to age of dwellings, λ_infilt_ is influenced by the types of dwellings that constitute the housing stock and by the climatic conditions (Persily et al. 2010). Parameters that influence λ_overall_ include CCDs, the fraction of time that cooling occurs (*x*), and the fraction of residences with central AC (*y*). Values for these parameters are listed in Table 1.

*Changes in indoor ozone and ozone-derived products.* When indoors, occupants are exposed to ozone transported from outdoors and ozone-derived products generated by the reaction of ozone with other indoor chemicals. For each 10-ppb increase in outdoor ozone, the Δ[O_3_]_in_ ranges from 1.3 to 2.0 ppb, whereas the Δ[prod]_in_ ranges from 2.4 to 2.6 ppb (Table 1). These results indicate that the indoor concentration of ozone varies more with the air change rate than the concentration of ozone-derived products does.

*Ozone exposure coefficient.* The estimated ozone exposure coefficient, ΔO_3_exposure_, ranges from 1.8 for Los Angeles to 2.5 for Boston (Table 1). Cities with lower CDD values tend to have a lower fraction of residences with central AC, but these cities did not necessarily have higher total ozone exposure. Total ozone exposure depends on additional factors such as the tightness of building envelopes and the fraction of time spent indoors.

**Figure 2:**
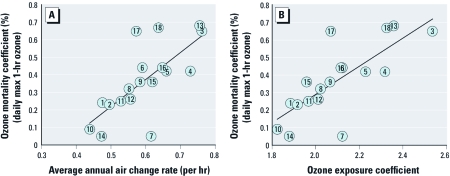
For the 18 NMMAPS cities for which detailed modeled infiltration rates were available, ozone mortality coefficients versus (*A*) average annual air change rates (*y* = 1.54*x* – 0.55, *R*^2^ = 0.51), and (*B*) ozone exposure coefficients (*y* = 0.81*x* – 1.32, *R*^2^ = 0.58). Ozone mortality coefficients based on daily maximum (max) 1-hr ozone. Numbers within circles refer to numbers listed in the first column of Table 1.

*Correlations.* We systematically explored potential correlations between selected parameters and ozone mortality coefficients using coefficients of determination (*R^2^*) and *p*-values from linear regressions. Figure 2A is a plot of ozone mortality coefficients, based on daily maximum 1-hr ozone, versus average annual overall air change rates. The least-squares linear regression has an *R^2^* of 0.51 and a *p*-value of < 0.001. Figure 2B is analogous, but for ozone mortality coefficients versus ozone exposure coefficients (*R^2^* = 0.58; *p*-value < 0.001). In general, results were similar but not as strong for ozone mortality coefficients based on 8-hr and 24-hr ozone metrics (see Table 2).

**Figure 3:**
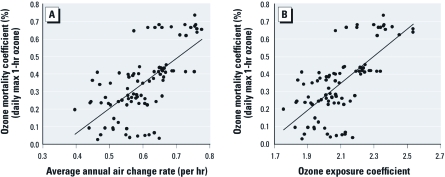
For all 90 NMMAPS cities included in the extended analysis, ozone mortality coefficients versus (*A*) average annual air change rates (1.42*x* – 0.50, *R*^2^ = 0.43), and (*B*) ozone exposure coefficients (*y* = 0.78*x* – 1.28, *R*^2^ = 0.47). Ozone mortality coefficients are based on daily maximum (max) 1-hr ozone.

*Exploring correlations for other NMMAPS cities.* As described in “Methods,” we selected an additional 72 NMMAPS cities for which we estimated overall air change rates and ozone exposure coefficients. Associations were reasonably strong for the larger set of 90 NMMAPS cities (the original 18 cities plus the 72 additional NMMAPS cities): air change rate: *R^2^* = 0.43, *p* < 001 (Figure 3a); ozone exposure coefficient: *R^2^* = 0.47, *p* < 001 (Figure 3b). Associations based on alternative ozone metrics for the 90 cities were consistent with those for the original set of 18 cities (Table 2).

*Sensitivity analysis.* Several parameters used in calculating average annual air change rates and ozone exposure coefficients are difficult to estimate. These include the fraction of year in cooling mode (*x*), the difference in air change rate when windows are open versus closed (λ_open_), the first-order rate constant for ozone removal by indoor surfaces (*k*_sr_), the fraction of residences with central AC (*y*), and the ratio of time indoors to outdoors (*t*_in_*/t*_out_). We examined the sensitivity of correlations between ozone mortality coefficients and ozone exposure coefficients when the latter are calculated with reasonable bounds for these input parameters. The selection criteria for the bounded estimates are presented in “Methods.” Table 3 summarizes results from the sensitivity analysis. The only parameter for which a bounded value meaningfully affected the results was the estimate for λ_open_. When we used a value of 5.0/hr (3.3 times larger than the default value), the association between the ozone exposure coefficient and ozone mortality coefficient was weaker (the *R^2^* range for the three ozone metrics was 0.27–0.34 compared with 0.48–0.58). However, using a value of 0.5/hr for λ_open_ (three times smaller than the default value) resulted in little effect on the association. The results were relatively insensitive to alternative values for *x*, *k*_sr_, *y*, or *t*_in_*/t*_out_.

## Discussion

*Infiltration rates, air change rates, and ozone exposure coefficients.* Cities with larger values for the fraction of year that cooling occurs (*x*) also tend to have a larger fraction of residences with central AC (*y*). Hence, the product “(*x*)(1–*y*)” in Equation 1 varies less from city to city than either of its constituent terms. The result is that λ_infilt_ and λ_overall_ have similarly strong correlations with ozone mortality coefficients. Associations are somewhat stronger for ΔO_3_exposure_ than for overall air change rates because the former account for city-to-city differences in the fraction of time spent outdoors.

*Effect modification of central and window AC.* Several studies have noted that ozone mortality coefficients tend to be lower in cities with a higher fraction of central AC (Bell and Dominici 2008; Levy et al. 2005; Medina-Ramon et al. 2006; Smith et al. 2009; Vedal 2009). Residential central AC systems typically are not designed to provide outdoor air; air change occurs primarily through infiltration (Persily et al. 2010; Smith et al. 2009; Weschler 2006). Consequently, residents in homes with central AC tend to be less exposed to indoor ozone of outdoor origin than residents in homes without central AC. We examined the relationship between ozone mortality coefficients and the fraction of homes with central AC and found that ozone mortality coefficients correlated much more strongly with ozone exposure coefficients than with the fraction of residences with central AC (Table 2). This indicates that the associations observed with total ozone exposure are determined by factors in addition to the fraction of residences with central AC, including the age and type of buildings, climate, and time-activity patterns.

Smith et al. (2009) found a weak inverse association between ozone mortality coefficients and central AC, and a strong positive association between ozone mortality and window AC. They speculated that outdoor-to-indoor transport might be higher in residences with window AC because of window opening in rooms without window AC units coupled with the option on many window units of opening a vent to introduce some outdoor air. Additionally, cities containing a larger fraction of residences with window AC have a larger fraction of older buildings, and older buildings tend to have higher infiltration rates than newer buildings, as noted above.

*Effects of seasonal variations and temperature.* Dominici et al. (2003) reported that median national ozone mortality coefficients are larger in the summer than in the winter (0.51% and –0.53%, respectively). Using Equation 4, we estimated average national ozone exposure coefficients for summer and winter, based on data for the 18 representative NMMAPS cities weighted for population, and found that the national average exposure coefficient was larger in the summer (2.36) than in the winter (2.01). [For details regarding these estimates, seeSupplemental Material, p. 9 (http://dx.doi.org/10.1289/ehp.1103970).]

Ren et al. (2008) used April–October data from 60 NMMAPS cities during 1987–2000 to examine how temperature influences the ozone mortality association in the eastern region of the United States. As part of this investigation, they estimated city-specific ozone mortality coefficients at low and high temperatures (defined as the lowest and highest tertiles of the temperature distribution of each city, respectively). They found that in the Northeast, ozone mortality coefficients were larger at high temperatures than at low temperatures; while in the Southeast, there was little change in ozone mortality coefficients across temperatures. Adopting the same stratification, we again used Equation 4 to calculate ozone exposure coefficients at high and low temperatures for the 13 eastern NMMAPS cities for which detailed infiltration rate distributions were available. In the Northeast, ozone exposure coefficients were larger at high temperatures than at low temperatures; while in the Southeast, the ozone exposure coefficients were closer in value at high and low temperatures [see Supplemental Material, Table 4 (http://dx.doi.org/10.1289/ehp.1103970)]. This finding is consistent with anticipated window opening during hot weather in homes without central AC, coupled with a larger fraction of homes being without central AC in the Northeast compared with the Southeast.

*Other effect modifiers.* Bell and Dominici (2008) and Smith et al. (2009) examined other factors that might influence ozone mortality coefficients among the NMMAPS cities (between-city effect modifiers). Both studies reported that greater use of public transportation was positively associated with ozone-related mortality, but Smith et al. (2009) also reported that communities with a larger proportion of residents who drove to work had lower ozone-related mortality. These results are consistent with expectations that ozone exposures are higher when using public transportation (more time outdoors) than commuting by car (less time outdoors). In addition, Smith et al. reported that communities with a high proportion of residents who had moved since 1995 had lower ozone mortality coefficients than other communities. We infer that such communities have lower annual air change rates, as they tend to have a larger fraction of new buildings.

*Limitations.* There are a number of limitations to the present analysis, beginning with the assumption that there is no threshold concentration for ozone’s impact on short-term mortality. Bell et al. (2006) reported that even low levels of ozone were associated with short-term mortality. However, from a physiological perspective, we would anticipate that, at higher ozone levels, antioxidants in the respiratory tract have a proportionately smaller impact on the amount of ozone that penetrates deep into the respiratory tract. Breathing rate also affects how deeply ozone penetrates the respiratory system, and the adverse health effects of ozone are anticipated to be greatest when ozone reaches the lungs (Lippmann 1989). Higher breathing rates typically occur outdoors, which was not taken into account in the present analysis.

We assumed that window opening only occurs in residences without central AC and only when cooling is required; we did not account for window opening during mild weather (in residences with or without central AC) or window opening as an alternative means of cooling in homes with central AC. Therefore, cities with extended periods of mild weather are likely to have larger average overall air change rates than assumed in the present study.

We lacked a well-matched representative city for NMMAPS cities located in the middle of the United States. These cities tend to have low ozone mortality coefficients and, based on the present analysis, would be anticipated to have low average air change rates. We do know that the fraction of residences with central AC, *y*, is high in these cities (e.g., Kansas City, KS, *y* = 0.84; Kansas City, MO, *y* = 0.84; Omaha, NE, *y* = 0.83; Wichita, KS, *y* = 0.80).

The infiltration rates used in the present analysis were for single family homes (Table 8 of Persily et al. 2010). We did not include infiltration rates for apartment buildings in our analysis as there are few measurements of airtightness and ventilation system performance in such buildings (Persily et al. 2010). We recognize that the proportion of residents who live in apartment buildings varies among cities and can be quite significant. This factor should be considered in future analyses as more data become available to characterize infiltration rates for apartment buildings in different geographic regions. Additionally, the infiltration rates for single-family homes likely do not apply to other indoor locations. NHAPS indicates that, on average, the U.S. population spends 69% of their time in residences, 5.4% in offices or factories, and 12.8% in other indoor locations (Klepeis et al. 2001).

Finally, our analysis ignored indoor sources of ozone such as photocopiers and ozone generators. We assumed that such indoor sources do not vary substantially from city to city.

## Conclusions

Although there are limitations with the present analysis, differences in ozone mortality coefficients among cities appear to partially reflect differences in ozone exposure coefficients resulting from differences in air change rates and time spent outdoors. The correlations are relatively robust over a reasonable range of estimates for the key input parameters.

The NMMAPS data used for the present analysis are for the period of 1987–2000. The housing stock data used for the modeled infiltration rate distributions were collected prior to 2001. Construction practices continue to change, and old buildings are being replaced by newer, tighter buildings. We anticipate that average annual infiltration rates have decreased over the last decade. It would be valuable to examine whether ozone mortality coefficients in U.S. cities have also decreased during this period. If so, this would provide further support for the association between ozone mortality coefficients and ozone exposure coefficients.

While reduced ventilation has the potential to decrease the indoor concentration of pollutants that originate outdoors, it increases the concentration of pollutants that originate indoors. A recent literature review addressing ventilation and human health concluded that increasing air change rates above current guidelines would reduce the prevalence of certain negative health effects (Sundell et al. 2011). Attempts to deliberately reduce ventilation with the aim of reducing total ozone exposure might have unintended adverse health consequences. A better solution would be to maintain adequate ventilation while removing ozone from ventilation air and controlling indoor sources.

## Supplemental Material

(512 KB) PDF
