# Supplementary material for: Assessing the Influence of Indoor Exposure to “Outdoor Ozone” on the Relationship between Ozone and Short-term Mortality in U.S. Communities
Source: Environ Health Perspect. 2011 Nov 18;120(2):235–40. doi: 10.1289/ehp.1103970 (PMC3279450; doi:10.1289/ehp.1103970)
Supplement: (512 KB) PDF [file ehp.1103970.s001.pdf]

Supplemental Material

Assessing the Influence of Indoor Exposure to "Outdoor Ozone" on the Relationship between Ozone and Short-term Mortality in U.S. Communities

Chun Chen[1], Bin Zhao[1], Charles J. Weschler[2,3*]

[1] Department of Building Science, School of Architecture, Tsinghua University, Beijing, 100084, China

[2] Environmental and Occupational Health Sciences Institute, UMDNJ–Robert Wood Johnson Medical School and Rutgers University, Piscataway, New Jersey, 08854, USA

[3] International Centre for Indoor Environment and Energy, Technical University of Denmark, Lyngby, Denmark

[*] Corresponding author

Table of Contents for Supplementary Materials — Page

Fraction of time cooling occurs 2

Average annual air change rate 2

Change in ozone-derived products 3

Ozone exposure coefficient 4

Supplemental Material, Table 1: Ozone mortality coefficients 5

Variation of ozone mortality coefficients with ozone metric 5

Supplemental Material, Table 2: Key input parameters and calculated results for all NMMAPS cities included in present study 6

Supplemental Material, Table 3: Default and upper bound ratios of time spent indoors to time outdoors for the targeted cities 9

Effects of seasonal variations and temperature 9

Supplemental Material, Table 4: Ozone mortality coefficients and ozone exposure coefficients at low and high temperatures for 13 eastern cities 10

References 11

Fraction of time cooling occurs

The fraction of time that cooling occurred in a given city (x) was estimated using a method based on the city's monthly maximum and minimum temperature, T_{max} and T_{min}, throughout a statistical year. The data used in this analysis were from NOAA (2002); the NOAA database is based on measurements made during the period from1971-2000. In this approach we assumed that cooling occurred if the temperature was higher than 24°C. A justification for this assumption is contained in the Residential Buildings Energy Consumption Survey (RECS) by the U.S. EIA (2005); this survey reports that the average temperature set-points of central AC in U.S. residential buildings during the day and night are approximately 23.5 ^{o}C and 24 ^{o}C, respectively. We further assumed that the daily temperature in a given month is uniformly distributed. Hence, for a given month, the fraction of time that cooling occurred was estimated as $(T_{max} – 24)/(T_{max} – T_{min})$. For example, if the maximum and minimum temperature in a given month is 30°C and 12°C, respectively, then "x" for this month would be 0.33. The values of "x" for each of the 12 months were averaged to give an average annual value for "x". The *Results* section examines the sensitivity of our results to "x", including estimates of lower and upper bounds for this parameter.

Average annual air change rate

The average annual air change rate for a given city ($\lambda_{overall}$) was estimated starting with its average annual infiltration rate (λ_{infilt}). These infiltration rates were derived assuming that windows in the residences were closed. Given that the housing stock includes residences with and without central AC, the reported infiltration rates in Persily et al. (2010) can be viewed as a combination of air change rates for residences with central AC when the AC systems are "on" or "off" and air change rates for residences without central AC:

$$\lambda_{infilt} = y[x\,\lambda_{AC\_on} + (1-x)\,\lambda_{AC\_off}] + (1-y)\,(\lambda_{win\_closed}) \qquad [S1]$$

where y is fraction of homes with central AC and $1-y$ is fraction without; x is the fraction of time that cooling occurs; λ_{AC_on} and λ_{AC_off} are the air change rates in residences with central AC when the AC is on or off, respectively; and λ_{win_closed} is the air change rate in residences without central AC when their windows are closed.

We are interested in the average air change rates in the different U.S. cities during periods when windows were open as well as when they were closed. We can algebraically express the method that we have used to estimate overall yearly average air change rates, $\lambda_{overall}$, as:

$$\lambda_{overall} = y[x\lambda_{AC\_on} + (1\text{-}x)\lambda_{AC\_off}] + (1\text{-}y)[(1\text{-}x)(\lambda_{win\_closed}) + x(\lambda_{win\_open})] \qquad [S2]$$

where λ_{win_open} is the air change rate in residences without central AC when their windows are open. Using the equivalency shown in Eq (S1), this simplifies to:

$$\lambda_{overall} = \lambda_{infilt} + (\mathrm{x})(1\text{-}y)[(\lambda_{win\_open}) - (\lambda_{win\_closed})] = \lambda_{infilt} + (\mathrm{x})(1\text{-}y)(\lambda_{open}) \qquad [1]$$

where $[(\lambda_{win\_open}) - (\lambda_{win\_closed})] = (\lambda_{open})$.

Change in ozone-derived products

Ozone reacts indoors to generate oxidation products. In most indoor settings, the volatile ozone-derived oxidation products are dominated by species that have originated as a consequence of surface- rather than gas-phase chemistry (Weschler 2006; Morrison 2008; Wisthaler and Weschler 2010). The change in the indoor gas-phase concentration of ozone derived products per 10 ppb change in outdoor ozone ($\Delta[prod]_{\mathrm{in}}$) can be approximated using Eq. S3 (Weschler, 2006):

$$\Delta[prod]_{\mathrm{in}} = (yld_g\,(k_{sr})/\lambda_{overall})\,\Delta[O_3]_{\mathrm{in}} \qquad [S3]$$

where yld_g is the average yield of gas phase products resulting from surface reactions and $\Delta[O_3]_{\mathrm{in}}$ is given by Eq. 2. Performing a substitution based on Eq. 2, this reduces to:

$$\Delta[prod]_{\mathrm{in}} = [(yld_g\,(k_{sr})/(\lambda_{\mathrm{overall}} + k_{sr})]\ 10\ \mathrm{ppb} \qquad [3]$$

Ozone exposure coefficient

Defining "exposure" in a given micro-environment as the product of "time spent in the micro-environment" and "pollutant concentration in the micro-environment during that time", the *change* in outdoor ozone exposure that results from a 10 ppb increase in outdoor ozone, $\Delta(outdr\ O_3\text{-}expos)$, is given by:

$$\Delta(outdr\ O_3\text{-}expos) = t_{out}\ (10\ \text{ppb}) \qquad [S4]$$

where t_{out}, is the time outdoors. The *change* in indoor ozone exposure that results from a 10 ppb increase in outdoor ozone, $\Delta(indr\ O_3\text{-}expos)$, is given by:

$$\Delta(indr\ O_3\text{-}expos) = t_{in}\ [\lambda_{\text{overall}}/(\lambda_{\text{overall}} + k_{sr})]\ (10\ \text{ppb}) \qquad [S5]$$

where t_{in}, is the time indoors, k_{sr} is the rate constant for ozone removal by indoor surfaces and $[\lambda_{\text{overall}}/(\lambda_{\text{overall}} + k_{sr})]$ estimates the indoor/outdoor ozone ratio (Weschler et al., 1989; Weschler, 2000; Weschler, 2006).

If we consider only "outdoor" and "indoor" environments, then the change in total ozone exposure resulting from a 10 ppb increase in outdoor ozone, $\Delta(total\ O_3\text{-}expos)$, is the sum of the change of exposures outdoors and indoors:

$$\Delta(total\ O_3\text{-}expos) = t_{out}\ (10\ \text{ppb}) + t_{in}\ [\lambda_{\text{overall}}/(\lambda_{\text{overall}} + k_{sr})]\ (10\ \text{ppb}) \qquad [S6]$$

More generally, the change in total ozone exposure per given change in outdoor ozone exposure, $\Delta O_{3\_\text{exposure}}$, is:

$$\begin{aligned}\Delta O_{3\_\text{exposure}} &= \Delta(total\ O_3\text{-}expos)/\Delta(outdr\ O_3\text{-}expos) \\ &= 1 + (t_{in}/t_{out})[\lambda_{\text{overall}}/(\lambda_{\text{overall}} + k_{sr})] \qquad [4]\end{aligned}$$

Throughout the paper we refer to $\Delta O_{3\_\text{exposure}}$ as the **ozone exposure coefficient**. Eq. 4 can be used to estimate the ozone exposure coefficient in different cities.

Supplemental Material, Table 1. For 18 representative cities, ozone mortality coefficients calculated using a 1-h ozone metric, an 8-h ozone metric or a 24-h ozone metric. In each case the ozone mortality coefficients are "posterior mean regional prior" estimates. Values have been taken from Figures 1, 4 and 5 in Smith et al., 2009.

#	City	Ozone mortality coefficient[a]		
		1-h [O_3]	8-h [O_3]	24-h [O_3]
1	Atlanta, GA	0.24	0.26	0.27
2	Birmingham, AL	0.23	0.28	0.21
3	Boston, MA	0.65	0.90	1.29
4	Buffalo, NY	0.42	0.57	0.68
5	Chicago, IL	0.42	0.73	0.89
6	Cincinnati, OH	0.44	0.70	1.06
7	Corpus Christi, TX	0.05	0.20	-0.03
8	Dallas/ Ft. Worth, TX	0.32	0.57	0.82
9	Denver, CO	0.36	0.03	-0.15
10	Los Angeles, CA	0.09	0.20	0.13
11	Miami, FL	0.25	0.32	0.36
12	Nashville, TN	0.26	0.36	0.53
13	NYC, NY	0.68	1.13	1.80
14	Phoenix, AZ	0.05	0.21	0.22
15	Seattle, WA	0.36	0.09	0.08
16	St. Louis, MO	0.44	0.69	0.72
17	Washington	0.65	0.92	1.48
18	Worcester, MA	0.67	1.04	1.54

[a] Coefficients correspond to the percent increase in short-term mortality per 10 ppb increase in outdoor ozone. In each case the values are "posterior mean regional prior estimates" from Figures 1, 4 and 5 of Smith et al. (2009).

Variation of ozone mortality coefficients with ozone metric

For the original 18 targeted cities, the association between ozone mortality coefficients and air change rates are stronger when the latter are based on the 1-h rather than 8-h ozone metric, or the 8-h rather than 24-h ozone metric (Table 2). The daily maximum 1-hr ozone level tends to occur during the warmest period of the day. This is also the period when the difference in air change rates among the various cities is likely to be most pronounced – that is, when the difference in air change rates between residences with central AC and residences without central AC are likely

largest (closed versus open windows). This approximate co-occurrence of daily maximum 1-h ozone and daily maximum difference in air change rate between residences with and without central AC may partially explain the stronger relationship observed when using mortality coefficients based on the daily maximum 1-h average. Such "co-occurrence" is less pronounced for the maximum 8-h average and is not a consideration for the 24-h average. This distinction is not observed for the correlations that have been extended to 90 cities, which may reflect greater uncertainties in estimated infiltration rates for 72 of these 90 cities.

Supplemental Material, Table 2. Key input parameters and calculated results for all NMMAPS cities included in the present study.

City	λ_{infilt}	*CDD*	*x*	*y*	$\lambda_{overall}$	$\Delta[O_3]_{in}$	$\Delta[prod]_{in}$	$\Delta O_{3\_exposure}$
	h^{-1}		[--]	[--]	h^{-1}	*ppb*	*ppb*	[--]
Akron, OH	0.61	679	0.08	0.38	0.69	1.9	2.4	2.27
Albuquerque, NM	0.49	1290	0.17	0.54	0.61	1.7	2.5	2.11
Arlington, VA	0.54	1560	0.14	0.64	0.62	1.7	2.5	2.13
Atlanta, GA	0.43	1810	0.22	0.86	0.48	1.4	2.6	1.89
Austin, TX	0.50	2974	0.35	0.91	0.55	1.5	2.5	2.02
Bakersfield, CA	0.42	2304	0.26	0.73	0.53	1.5	2.6	1.97
Baltimore, MD	0.54	1220	0.22	0.64	0.66	1.8	2.5	2.20
Baton Rouge, LA	0.50	2652	0.31	0.77	0.61	1.7	2.5	2.11
Biddeford, ME	0.68	347	0.00	0.18	0.69	1.9	2.4	2.41
Birmingham, AL	0.43	1881	0.22	0.80	0.50	1.4	2.6	1.92
Boston, MA	0.68	777	0.07	0.18	0.76	2.0	2.4	2.54
Buffalo, NY	0.70	548	0.03	0.43	0.73	2.0	2.4	2.32
Charlotte, NC	0.54	1644	0.21	0.79	0.61	1.7	2.5	2.09
Chicago, IL	0.61	835	0.07	0.51	0.66	1.8	2.5	2.23
Cincinnati, OH	0.52	1210	0.11	0.57	0.59	1.6	2.5	2.12
Cleveland, OH	0.61	712	0.05	0.43	0.66	1.8	2.5	2.23
Colorado Spg, CO	0.49	443	0.06	0.18	0.56	1.6	2.5	2.03
Columbus, GA	0.43	925	0.28	0.86	0.49	1.4	2.6	1.91
Columbus, OH	0.61	2297	0.11	0.73	0.65	1.8	2.5	2.22
Corpus Christi, TX	0.48	3498	0.41	0.78	0.62	1.7	2.5	2.13
Coventry, RI	0.68	714	0.03	0.19	0.71	1.9	2.4	2.45
Dallas/Ft Wth, TX	0.50	2571	0.32	0.89	0.55	1.6	2.5	2.03
Dayton, OH	0.61	935	0.09	0.38	0.69	1.9	2.4	2.28
Denver, CO	0.49	695	0.09	0.32	0.58	1.6	2.5	2.07
Detroit, MI	0.61	727	0.09	0.57	0.67	1.8	2.5	2.25
El Paso, TX	0.50	2165	0.29	0.81	0.58	1.6	2.5	2.07
Evansville, IN	0.52	1422	0.17	0.79	0.57	1.6	2.5	2.10

Fort Wayne, IN	0.61	830	0.08	0.52	0.67	1.8	2.5	2.24
Fresno, CA	0.42	1991	0.23	0.80	0.49	1.4	2.6	1.90
Grand Rapids, MI	0.61	613	0.06	0.52	0.65	1.8	2.5	2.22
Greensboro, NC	0.54	1332	0.16	0.79	0.59	1.6	2.5	2.06
Houston, TX	0.50	2893	0.35	0.84	0.58	1.6	2.5	2.07
Huntsville, AL	0.51	1671	0.20	0.80	0.57	1.6	2.5	2.04
Indianapolis, IN	0.52	1042	0.11	0.78	0.56	1.6	2.5	2.07
Jackson, MS	0.43	2290	0.25	0.65	0.57	1.6	2.5	2.03
Jacksonville, FL	0.43	2636	0.31	0.84	0.50	1.4	2.6	1.93
Jersey City, NJ	0.62	882	0.08	0.14	0.73	2.0	2.4	2.32
Johnstown, PA	0.70	803	0.10	0.48	0.78	2.1	2.4	2.37
Kingston, NY	0.62	220	0.02	0.20	0.65	1.8	2.5	2.20
Knoxville, TN	0.51	1450	0.16	0.81	0.56	1.6	2.5	2.01
Lafayette, LA	0.50	2671	0.33	0.77	0.61	1.7	2.5	2.12
Lake Charles, LA	0.50	2705	0.34	0.84	0.58	1.6	2.5	2.07
Las Vegas, NV	0.42	3214	0.37	0.92	0.46	1.3	2.6	1.87
Lexington, KY	0.51	1154	0.13	0.68	0.57	1.6	2.5	2.03
Little Rock, AR	0.50	2086	0.25	0.86	0.55	1.6	2.5	2.02
Los Angeles, CA	0.42	1506	0.02	0.34	0.44	1.3	2.6	1.83
Louisville, KY	0.51	1443	0.16	0.68	0.59	1.6	2.5	2.06
Madison, WI	0.61	582	0.05	0.45	0.66	1.8	2.5	2.22
Memphis, TN	0.51	2190	0.25	0.78	0.60	1.7	2.5	2.07
Miami, FL	0.35	4383	0.59	0.80	0.53	1.5	2.6	1.97
Milwaukee, WI	0.61	616	0.05	0.50	0.65	1.8	2.5	2.21
Mobile, AL	0.43	2548	0.30	0.76	0.54	1.5	2.5	1.98
Modesto, CA	0.42	1570	0.19	0.90	0.45	1.3	2.6	1.84
Muskegon, MI	0.61	487	0.03	0.52	0.63	1.7	2.5	2.19
Nashville, TN	0.51	1656	0.18	0.83	0.56	1.6	2.5	2.01
New Orleans, LA	0.48	2776	0.35	0.70	0.64	1.8	2.5	2.16
New York, NY	0.62	1160	0.10	0.10	0.76	2.0	2.4	2.36
Newark, NJ	0.62	1220	0.12	0.25	0.76	2.0	2.4	2.36
Oakland, CA	0.42	530	0.04	0.27	0.46	1.3	2.6	1.87
Ok. City, OK	0.50	1907	0.22	0.81	0.56	1.6	2.5	2.04
Orlando, FL	0.35	3457	0.42	0.93	0.40	1.2	2.6	1.76
Philadelphia, PA	0.62	1235	0.13	0.47	0.73	2.0	2.4	2.30
Phoenix, AZ	0.42	4189	0.44	0.92	0.47	1.4	2.6	1.88
Pittsburgh, PA	0.70	726	0.07	0.48	0.75	2.0	2.4	2.34
Portland, OR	0.62	398	0.03	0.06	0.66	1.8	2.5	2.01
Providence, RI	0.68	714	0.07	0.19	0.76	2.0	2.4	2.54
Raleigh, NC	0.54	1521	0.18	0.85	0.58	1.6	2.5	2.05
Riverside, CA	0.42	1697	0.22	0.73	0.51	1.5	2.6	1.94
Rochester, NY	0.70	577	0.04	0.35	0.74	2.0	2.4	2.34
Sacramento, CA	0.42	1248	0.17	0.80	0.47	1.4	2.6	1.88
Salt Lake City, UT	0.49	1089	0.12	0.38	0.60	1.7	2.5	2.09
San Antonio, TX	0.48	3038	0.36	0.69	0.65	1.8	2.5	2.17

SanBernrdino, CA	0.42	1937	0.23	0.73	0.51	1.5	2.6	1.94
San Diego, CA	0.42	866	0.09	0.30	0.51	1.5	2.6	1.94
San Jose, CA	0.42	811	0.11	0.31	0.53	1.5	2.5	1.97
Santa Anna, CA	0.42	1294	0.13	0.34	0.55	1.6	2.5	2.00
Seattle, WA	0.62	192	0.00	0.06	0.62	1.7	2.5	1.96
Shreveport, LA	0.50	2396	0.30	0.65	0.66	1.8	2.5	2.18
Spokane, WA	0.62	394	0.04	0.37	0.66	1.8	2.5	2.01
St. Louis, MO	0.58	1561	0.23	0.80	0.65	1.8	2.5	2.12
St. Petersburg, FL	0.35	3718	0.47	0.87	0.45	1.3	2.6	1.84
Stockton, CA	0.42	1456	0.19	0.71	0.50	1.4	2.6	1.93
Syracuse, NY	0.70	551	0.05	0.20	0.76	2.0	2.4	2.36
Tacoma, WA	0.62	167	0.01	0.07	0.63	1.7	2.5	1.98
Tampa, FL	0.35	3481	0.46	0.87	0.44	1.3	2.6	1.83
Toledo, OH	0.61	715	0.07	0.50	0.66	1.8	2.5	2.24
Tucson, AZ	0.42	3017	0.40	0.65	0.63	1.7	2.5	2.13
Tulsa, OK	0.50	2049	0.25	0.82	0.57	1.6	2.5	2.05
Washington, DC	0.54	1560	0.12	0.82	0.57	1.6	2.5	2.07
Worcester, MA	0.60	371	0.03	0.10	0.64	1.8	2.5	2.33

Abbreviations: λ_{infilt}, infiltration rates; *CDD*, cooling degree days; *x*, fraction of year cooling occurs; *y*, fraction of residences with central AC; $\lambda_{overall}$, overall air change rate; $\Delta[O_3]_{in}$, change in indoor ozone concentration per 10 ppb change in outdoor ozone concentration; $\Delta[\text{prod}]_{in}$, change in indoor concentration of ozone-derived products per 10 ppb change in outdoor ozone concentration; $\Delta O_{3\_exposure}$, change in total ozone exposure per unit change in outdoor ozone exposure, referred to as the "ozone exposure coefficient".

Supplemental Material, Table 3. Default and upper bound ratios of time spent indoors to time outdoors, t_{in}/t_{out}, for the targeted cities.

**City**	t_{in}/t_{out}**, default**[a]	t_{in}/t_{out}**, upper bound**[b]
	[--]	*[--]*
Atlanta, GA	6.47	12.0
Birmingham, AL	6.47	12.0
Boston, MA	7.58	16.4
Buffalo, NY	6.74	12.9
Chicago, IL	6.83	13.7
Cincinnati, OH	6.83	13.7
Corpus Christi, TX	6.58	12.9
Dallas/Ft. Wrth, TX	6.58	12.9
Denver, CO	6.54	10.9
Los Angeles, CA	6.46	11.9
Miami, FL	6.47	12.0
Nashville, TN	6.47	12.0
New York City, NY	6.74	12.9
Phoenix, AZ	6.46	11.9
Seattle, WA	5.61	8.86
St. Louis, MO	6.27	11.3
Washington, DC	6.66	11.9
Worcester, MA	7.58	16.4

[a] Time spent in vehicles treated as time outdoors.

[b] Time spent in vehicles treated as time indoors.

Effects of seasonal variations and temperature

Ozone mortality coefficients are known to vary seasonally and geographically (Bell et al., 2005; Dominici et al., 2003; Gryparis et al., 2004; Ito et al., 2005; Levy et al., 2005; Ren et al., 2008). As a further test of the associations reported in this paper, we have examined if seasonal differences in ozone mortality coefficients vary in the anticipated manner with seasonal differences in ozone exposure coefficients. We have not been able to find seasonal ozone mortality coefficients for the NMMAPS cities in the published literature. However, median values of national average ozone mortality coefficients for summer (June, July, August) and winter (December, January, February) have been reported in Figure 13 of Dominici et al., 2003 (summer, 0.51%; winter, -0.53%). For a given city, monthly values for ozone exposure coefficients can be calculated using Equation 4 and the approach described in the *Ozone exposure coefficients* subsection of the *Methods* section. More specifically, monthly values for

$\lambda_{overall}$ were calculated for each of the 18 cities. These, in turn, were calculated using Equation 1 and monthly values of x for each of the cities. From these monthly ozone exposure coefficients for the 18 representative NMMAPS cities, weighted for population, average national ozone exposure coefficients were calculated for summer and winter months.

Supplemental Material, Table 4. Ozone mortality coefficients and ozone exposure coefficients at low and high temperatures for 13 eastern NMMAPS cities.

	Ozone mortality coefficient [a] [%]		Ozone exposure coefficients [-]	
	Low temp.	High temp.	Low temp.	High temp.
Boston, MA	2.54	6.32	2.40	3.23
Buffalo, NY	2.49	5.39	2.28	2.55
Chicago, IL	1.66	9.21	2.15	2.65
Cincinnati, OH	1.48	6.18	2.01	2.72
New York City, NY	3.91	10.7	2.15	3.31
St. Louis, MO	1.74	6.52	2.02	2.42
Washington	2.83	5.87	2.02	2.36
Worcester, MA	0.22	5.00	2.26	2.68
Northeast	2.82	8.66	2.15 [b]	2.94[b]
Atlanta, GA	4.13	3.70	1.81	2.14
Birmingham, AL	1.52	2.39	1.81	2.27
Dallas/Ft Worth, TX	2.61	2.39	1.94	2.19
Miami, FL	9.35	-0.22	1.92	2.15
Nashville, TN	-0.43	1.96	1.94	2.31
Southeast	4.69	2.82	1.89 [c]	2.15[c]

[a] Mean value taken from Figure 2 of Ren et al. (2008), which displays “percent increase in daily mortality per 10 ppb increase in three-day moving average of ozone (log relative risk) with Bayesian estimates for specific communities by temperature levels.”

[b] Obtained by population-weighted averaging ozone exposure coefficients of the 8 representative Northeast cities.

[c] Obtained by population-weighted averaging ozone exposure coefficients of the 5 representative Southeast cities.

References

Bell ML, Dominici F, Samet JM. 2005. A meta-analysis of time-series studies of ozone and mortality with comparison to the national morbidity, mortality, and air pollution study. Epidemiology 16:436-445.

Bell ML, Dominici F. 2008. Effect modification by community characteristics on the short-term effects of ozone exposure and mortality in 98 US communities. American Journal of Epidemiology 167:986-997.

Dominici F, McDermott A, Daniels M, Zeger SL and Samet JM 2003a. Mortality among residents of 90 cities. In Revised Analyses of the National Morbidity, Mortality and Air Pollution Study, Part II. Health Effects Institute, Cambridge, MA, pp. 9–24.

Gryparis A, Forsberg B, Katsouyanni K, Analitis A, Touloumi G, Schwartz J, et al. 2004. Acute effects of ozone on mortality from the "Air pollution and health: A European approach" project. American Journal of Respiratory and Critical Care Medicine 170:1080-1087.

Ito K, De Leon SF, Lippmann M. 2005. Associations between ozone and daily mortality: analysis and meta-analysis. Epidemiology 16:446-457.

Klepeis NE, Nelson WC, Ott WR, Robinson JP, Tsang AM, Switzer P, et al. 2001. The National Human Activity Pattern Survey (NHAPS): a resource for assessing exposure to environmental pollutants. J Expo Anal Environ Epidemiol 11:231-252.

Levy JI, Chemerynski SM, Sarnat JA. 2005. Ozone exposure and mortality: an empiric bayesmetaregression analysis. Epidemiology 16:458-468.

Morrison G. 2008. Interfacial chemistry in indoor environments. Environ Sci Tech 42:3494-3499.

NOAA 2002. "United States Climate Normals 1971-2000. Annual Degree Days to Selected Bases." Climatography of the United States No. 81. Supplement No. 2. National Oceanic and Atmospheric Administration, Asheville, NC.

Persily A, Musser A, Emmerich SJ. 2010. Modeled infiltration rate distributions for U.S. housing. Indoor Air 20:473-485.

Persily A, Musser A, Emmerich SJ. 2011. Ventilation rate frequency distributions for U.S. housing stock. Report PWS NB863060-7-03758, National Institute of Standards and Technology. Gaithersburg, MD.

Ren C, Williams GM, Mengersen K, Morawska L, Tong S. 2008. Does temperature modify short-term effects of ozone on total mortality in 60 large eastern US communities? — An assessment using the NMMAPS data. Environ International 34: 451-458.

Smith RL, Xu BW, Switzer P. 2009. Reassessing the relationship between ozone and short-term mortality in US urban communities. Inhal Toxicol 21:37-61.

U.S. Energy Information Administration (EIA) 2005.Residential Buildings Energy Consumption Survey (RECS) Public Use Data. Available: http://www.eia.doe.gov/emeu/cbecs/public_use.html) [accessed 8 August 2011].

Weschler CJ, Shields HC, Naik DV. 1989. Indoor ozone exposures. J Air Pollut Control Assoc 39:1562-1568.

Weschler CJ. 2000. Ozone in indoor environments: Concentration and chemistry. Indoor Air 10:269-288.

Weschler CJ. 2006. Ozone's impact on public health: Contributions from indoor exposures to ozone and products of ozone-initiated chemistry. Environ Health Perspect 114:1489-1496.

Wisthaler A, Weschler CJ. 2010. Reactions of ozone with human skin lipids: sources of carbonyls, dicarbonyls, and hydroxycarbonyls in indoor air. Proc Natl Acad Sci 107:6568-6575.
